# Meeting report of the 2nd German dialectical behavior therapy for adolescents network meeting

**DOI:** 10.1186/s12919-016-0003-3

**Published:** 2016-07-04

**Authors:** Florian Hammerle, Arne Bürger, Michael Kaess, David R. Kolar

**Affiliations:** Department of Child and Adolescent Psychiatry and Psychotherapy, University Medicine Mainz, Langenbeckstrasse 1, 55131 Mainz, Germany; Department of Child and Adolescent Psychiatry, Psychosomatics and Psychotherapy, University of Wuerzburg, Würzburg, Germany; Department of Child and Adolescent Psychiatry, University Hospital Heidelberg, Heidelberg, Germany

## Abstract

Over the past 30 years, dialectical behavior therapy has been shown to be an effective treatment for adult borderline personality disorder. The adaptation of DBT for adolescents (DBT-A) in different patient groups has also led to some promising improvements of the respective psychopathology. During the second German DBT-A network meeting in 2015 in Mainz, Germany, a need for further research and innovative approaches in treatment of adolescents became apparent and resulted in controversial discussions. Main issues were enlarging evidence of effectiveness of DBT-A strategies with regard to family interaction, i.e. involving caregivers in treatment. In general, there seems to be a dire need for disentangling different therapeutic strategies and resulting treatment outcomes, especially concerning the needs of different patient groups. Additionally, the implementation of smartphone-based real life assessment and intervention into DBT-A was discussed extensively. Providing time congruent skills within an application, decreasing aversive tension and reducing dysfunctional behavior could lead to an enhanced therapist-patient interaction. This meeting report presents the core issues raised during the network meeting and discusses their implications for further research.

## Introduction

The second German Dialectical Behavior Therapy for Adolescents (DBT-A) network meeting was held on 6th and 7th of November 2015, in Mainz, Germany by the Department of Child and Adolescent Psychiatry and Psychotherapy of the University Medicine Mainz, in collaboration with the German head organization for DBT (DDBT). The DBT-A network meeting hosted 73 mental healthcare professionals from Germany, Austria, Switzerland and Luxemburg. In this meeting report, a short summary of the conference plenaries will be given, further topics of the conference research group will be outlined and the challenges for further research in DBT-A as identified will be described.

## Discussion

### State-of-the-art DBT-A in Germany

Over two days, several state-of-the-art presentations were given at the plenaries. After the opening ceremonies, Kristin von Auer presented state-of-the-art developments in DBT-A with special reference to advances in individual psychotherapy and the development of a new version of adolescent skills-training in Germany [1, 2]. Three new modules (walking the middle path, and for the first time modules providing interpersonal effectiveness skills and skills to improve self-worth) were added to the manual and their possible clinical applications were discussed in the plenary. Michael Kaess concluded the first day with a plenary lecture concerning the importance of non-suicidal self-injury (NSSI) and various other risk-taking and self-damaging behaviors as potential markers for early identification of adolescents with mental health problems, e.g. with emerging personality disorder [3]. On the second day, Florian Hammerle and Arne Bürger presented new approaches in the treatment of eating disorders based on recent findings of increased aversive tension in adolescents with eating disorders [4], and leading to the integration of adapted DBT-hierarchy, including life-threatening underweight in addition to suicidal behavior as a primary treatment aim for patients with anorexia nervosa [5, 6]. The plenaries section was concluded by Andrea Dixius, who presented network aspects between inpatient and outpatient DBT-A treatment in a German clinic compound, providing improved health care when using a close collaboration between different therapy stages. Special focus was put on the implementation of DBT-A at all stages even in subsequent rehabilitation or foster care to guarantee a continued treatment of adolescents with BPD or severe emotion regulation difficulties. After the plenaries section, work groups for DBT-A inpatient treatment, DBT-A outpatient treatment, DBT-A trauma therapy and DBT-A research were formed and their conclusions were presented in plenary.

### Results of the research work group: challenges for further research in the field of DBT-A

In Germany, several small pilot studies have been conducted on DBT-A [7, 8]. In addition, one larger trial compared cognitive behavior therapy and DBT-A with anorexia or bulimia nervosa [9]. Given the prominence of DBT-A in German-speaking countries and its widespread distribution in both inpatient and outpatient treatment facilities, there is a large potential for further high quality research. Hence, the research work group of the meeting identified several key areas of DBT-A that warrant further investigation.

#### Effectiveness of DBT-A

Since the original publication of adapting core DBT strategies and skills for adolescents [10], DBT-A has been frequently used and constantly updated [11, 12]. There are an increasing number of studies with adolescents using DBT strategies in both individual therapy and skills training. However, most research relies on open and quasi-experimental designs, whereas strong evidence derived from randomized controlled trials (RCT) is scarce [7, 11, 13, 14]. DBT has been further adapted for various disorders (borderline personality disorder, externalizing disorders, eating disorders and PTSD) and isolated problematic behaviors (NSSI, suicidal behavior) [8, 15–18]. While there is now first evidence for overall effectiveness of DBT-A for self-harming adolescents [19], there is a lack of knowledge if DBT strategies and skills available are equally useful for different forms of psychopathology in different age groups. Therefore, further research should initially concern the overall effectiveness of DBT strategies in adolescents.

Caregivers are typically involved in the treatment of adolescents. DBT-A integrates caregivers and family members within the first stages of therapy (i.e. when addressing commitment issues) and further throughout the treatment. This overall involvement of caregivers (i.e. mostly family members) as a typical component of DBT-A has not been investigated thus far. As caregivers are involved in both individual therapy and group skills training, this seems to be a very important research issue.

#### What works for whom? – disentangling DBT-A

On a most basic level, only very few studies in adults comprise differential analysis of individual therapy versus skills training and combined programs [20]. In contrast to adult DBT, skills training for adolescents include additive modules (walking the middle path) and teach both adolescents and caregivers together [11]. Several studies indicate that DBT-A skills training might be an effective treatment regardless of whether it is accompanied by individual therapy [21]. Hence, analysis of DBT-A treatment effectiveness and efficacy should go beyond overall effectiveness of therapeutic strategies, towards investigating differential effects of individual therapy versus skills training and determining important differences between treatment in adolescents [12, 22].

Strategies as first described by M. Linehan [23] include core cognitive behavioral strategies (shaping/chaining of behavior, cognitive interventions) and expand these by including a hierarchy of problem behavior, validation, commitment and dialectical strategies [24, 25]. No research has addressed differential effectiveness of central DBT strategies (e.g. dialectical strategies) and the evaluation versus cognitive behavioral strategies (e.g. socratic questioning).

Reduction in aversive tension may depend on accuracy (e.g. using distracting distress tolerance skills only when experiencing high levels of aversive tension), frequency and contingency of application of skills. These aspects have been investigated with regard to levels of practicing skills in adults [26], whereas studies scrutinizing connections between differential patterns of skill practice or frequency and reduction in aversive tension are missing. According to the principle of what works for whom, there seems to be a dire need for dismantling and component studies disentangling the effectiveness of single therapy strategies, particularly concerning different disorders in adolescents [27].

#### Using smartphones to enhance DBT-A

Standard DBT-A consists of weekly individual therapy sessions accompanied by group skills training, in which participants are taught several skills to manage states of high aversive tension, reduce problem behavior (e.g. NSSI, substance abuse), build up their interpersonal competence and generally increase their quality of life [12]. Participants are encouraged to apply their newly learned skills in their daily routine and success is controlled in individual sessions by the revision of paper-based diary cards. Additionally, telephone coaching for crisis and skills teaching in daily life is offered by most DBT therapists.

Given the widespread distribution of smartphones among adolescents, DBT-A mobile applications (apps) are a promising tool for therapist-patient interaction in real life. Several of the DBT-A key features could be integrated into a smartphone app to enhance the effectiveness of the treatment. For example, DBT-A apps could implement diary cards and telephone coaching. As most smartphone users have a mobile data plan or are frequently using wireless network connections, a direct upload function of diary cards to the therapist could enhance individual DBT sessions as the reviewing of diary cards can be undertaken in advance to the treatment session. Additionally, an innovative DBT-A app could integrate new features such as audiovisual repetition of previously learned skills, text-based coaching and individual skill presentation dependent on the current emotional state.

There are already some smartphone apps focusing on DBT available in prominent app markets, but only one app was used in a RCT [28]. Unfortunately, most apps do not allow a therapist-patient interaction from within the app and only few provide audiovisual material. Therefore, the development of a DBT app and the verification of its efficacy in a randomized controlled trial is an idea worth considering for future research.

## Conclusions

More than a decade ago, the concept of DBT-A was established for the first time in Germany. Further treatment centers are working to implement DBT-A into their clinical routine. The second German DBT-A network meeting reflected the importance of DBT-A for clinical practitioners and the interest in further developing the treatment.

Several important implications for further research were given by the conference research work group. There is a clear need for dismantle studies disentangling the effectiveness of specific DBT-A features (e.g. family involvement or skills teaching) in order to design more detailed and evidence-based DBT-A treatment approaches tailored to different patient groups. Additionally, DBT-A should focus on the potential of mobile apps for treatment enhancement. Future randomized controlled trials implementing innovative DBT-A mobile apps in treatment are highly welcomed and could provide practitioners with another tool for a more effective treatment.
